# Cross-linguistic differences in parafoveal semantic and orthographic processing

**DOI:** 10.3758/s13414-021-02329-7

**Published:** 2021-07-26

**Authors:** Leigh B. Fernandez, Christoph Scheepers, Shanley E. M. Allen

**Affiliations:** 1 University of Kaiserslautern, Erwin-Schrödinger-Straße, Building 57, 67663 Kaiserslautern, Germany; 2 University of Glasgow, Glasgow, UK

**Keywords:** Eye movements and reading, Parafoveal processing, Cross-linguistic influence, Bilingual word recognition

## Abstract

In this study we investigated parafoveal processing by L1 and late L2 speakers of English (L1 German) while reading in English. We hypothesized that L2ers would make use of semantic and orthographic information parafoveally*.* Using the gaze contingent boundary paradigm, we manipulated six parafoveal masks in a sentence (Mark found th*e wood for the fire; * indicates the invisible boundary): identical word mask (*wood*), English orthographic mask (*wook*), English string mask (*zwwl*), German mask (*holz*), German orthographic mask (*holn*), and German string mask (*kxfs*). We found an orthographic benefit for L1ers and L2ers when the mask was orthographically related to the target word (*wood* vs. *wook*) in line with previous L1 research. English L2ers did not derive a benefit (rather an interference) when a non-cognate translation mask from their L1 was used (*wood* vs. *holz*), but did derive a benefit from a German orthographic mask (*wood* vs. *holn)*. While unexpected, it may be that L2ers incur a switching cost when the complete German word is presented parafoveally, and derive a benefit by keeping both lexicons active when a partial German word is presented parafoveally (narrowing down lexical candidates). To the authors’ knowledge there is no mention of parafoveal processing in any model of L2 processing/reading, and the current study provides the first evidence for a parafoveal non-cognate orthographic benefit (but only with partial orthographic overlap) in sentence reading for L2ers. We discuss how these findings fit into the framework of bilingual word recognition theories.

## Introduction

During reading, we make a series of rapid eye movements (called saccades) that are interjected with moments in time (called fixations) where the eyes remain relatively still in order to take in information. Due to saccadic suppression, hardly any information about the text is accessed during saccades, so most meaningful information processing takes place during fixations (Matin, 1974). Importantly for reading, fixations encompass the foveal and parafoveal area of the visual field. The foveal area includes the central 2° of visual angle, and this is where visual acuity is highest (Schotter et al., 2012). The parafoveal area extends to 5° of visual angle. Although visual acuity decreases outside of the foveal area, the parafoveal area is still important during reading since it allows us to pre-process information that we are not directly fixating on, as well as plan upcoming eye movements (e.g., Rayner, 1998).

**Fig. 1:**
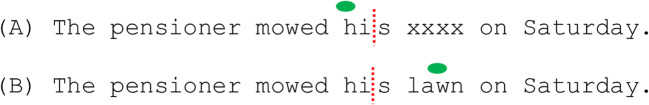
Illustration of the Gaze Contingent Boundary (GCB) paradigm. The green ellipses represent hypothetical fixation points and the red dotted lines represent a pre-defined boundary that is invisible to the reader. As soon as the eye crosses the invisible boundary, the mask “xxxx” in (**a**) is permanently replaced with the critical word “lawn” in (**b**)

The type of information that can be processed in the parafoveal region has traditionally been tested using the Gaze Contingent Boundary paradigm (GCB; Rayner, 1975). In this paradigm, a critical word in the text is initially blocked out with a mask, such as xxxx in the example below (Fig. 1). Critically, as soon as the reader performs a saccade that crosses an invisible boundary (indicated by red dotted lines in Fig. 1), the mask is permanently replaced with the critical word (here, lawn).

Due to saccadic suppression, readers typically remain unaware of this display change (Matin, 1974), and if the invisible boundary is set close to the critical word position (usually within four characters to the left of the critical word’s first character), it becomes possible to study parafoveal processing of the critical word. Specifically, by comparing conditions where the word is initially masked (Fig. 1) with conditions where it is not (i.e., displaying the word lawn instead of the mask in Fig. 1a), we can study whether reading behaviors on the critical word lawn differ when it is parafoveally unavailable (Fig. 1) relative to when it is parafoveally available (unmasked condition). The mask need not be uninformative, as in the example above, but can share features with the critical word. For instance, a mask like lawx would share orthographic information with the critical word (the first three letters are the same), while a mask like turf would share semantic and lexicality features with the critical word lawn.

Using the GCB paradigm, it has been found that readers are not only able to process the word that is currently fixated, but also the word in the parafoveal area of that fixation. For example, relative to a no-mask condition, uninformative masks (like xxxx in Fig. 1) lead to interference when fixating the critical word; relative to an uninformative mask, an orthographically related mask (e.g., lawx) can facilitate reading of the critical word. This is known as the *N+1 preview effect* (Vasilev & Angele, 2017), with N referring to the word in the foveal area and +1 referring to the subsequent (critical) word in the parafoveal area of fixating word N. The type of parafoveal mask can impact the reading of the critical word and can lead to an N+1 benefit or to N+1 interference (or a combination; see Kliegl et al., 2013) when processing the previously unavailable N+1 word (i.e., critical word).

An N+1 preview benefit has been found for masks that share orthographic, phonological, and in some languages morphological information with the critical word (see Schotter et al., 2012 for a review). However, for masks that share semantic information, the results are mixed and seem to arise only for specific languages, writing systems, or only under certain circumstances. For example, Rayner et al. (1986) tested whether reading a critical N+1 word was facilitated when parafoveally masked by a semantically related English word. Participants read sentences like My brother has brilliantly composed a ne*w *song* *for the school play* (where the critical word is underscored and * indicates the position of the invisible boundary). The critical word *song* was either parafoveally available (no display change) or masked with, respectively, a semantically related word (*tune*), an unrelated word (*door*), or an orthographically similar nonword (*sorp*). Surprisingly, they found that, relative to no display-change condition, gaze durations on the critical word were *similar* when orthographically similar masks were used (*sorp*) but were *greater* when semantically related (*tune*) or unrelated (*door*) masks were used, with no clear difference between the two conditions. This suggests that semantically related masks do not elicit an N+1 preview benefit. More recently, Rayner et al. (2014) replicated this study and found, again, no evidence for a semantic N+1 benefit. This lack of parafoveal semantic facilitation has been found in several studies with alphabetical languages (e.g., Finnish – Hyönä & Häikiö, 2005; English – Rayner et al., 1980; Spanish/English bilinguals – Altarriba et al., 2001).

However, other studies have indeed shown some semantic N+1 preview benefit in English. For example, Schotter (2013) found an N+1 preview benefit when the parafoveal mask and critical word were actual synonyms of one another (e.g., *curlers* / *rollers*) but not when they were mere semantic associates (e.g., *curlers* / *styling*). More evidence for semantic facilitation has been found when individual differences were taken into account; Veldre and Andrews (2016) showed that readers who scored high on the Nelson-Denny reading test (a measure of reading ability; Brown et al., 1993) were more efficient at extracting parafoveal semantic information than lower scorers. A larger semantic N+1 preview benefit in English has also been observed when the first letter of the critical N+1 word was capitalized relative to when it was not, suggesting that the visual salience of the capitalized letter may increase attention to the parafoveal word and thus increase the likelihood of semantic information extraction (Rayner & Schotter, 2014).

One potential reason for the semantic preview benefit being relatively hard to detect in English is the deep orthography of the language. Deep orthography languages have an opaque spelling to sound correspondence, while shallow orthography languages have a more straightforward spelling to sound correspondence. It may be that in languages with deep orthography, phonological decoding requires more cognitive resources relative to languages with a relatively shallow orthography, thus leaving fewer cognitive resources for parafoveal pre-processing of semantic information from word N+1. Indeed, this seems to be the case in comparison to German, which has a relatively clear spelling-to-sound correspondence. In both a parafoveal fast-priming study and a GCB study, Hohenstein and colleagues (Hohenstein et al., 2010; Hohenstein & Kliegl, 2014) found that German speakers were clearly able to make use of parafoveally previewed semantic information. Specifically, German speakers displayed facilitated reading of a critical word such as *Knochen* (bone(s)) when it was parafoveally masked with a semantically related word such as *Schädel* (skull(s)) compared to when it was parafoveally masked with a semantically unrelated word such as *Stiefel* (boot(s)).

Chinese uses a logographic writing system where the orthography of a word maps closely onto its meaning. Compared to alphabetical writing systems, fixations on word N tend to be much closer to word N+1 in Chinese, because words typically comprise only one to two characters in Chinese and inter-word spaces are generally absent. This means that readers of Chinese should have more parafoveal information available (a) due to higher visual acuity for parafoveal words and (b) due to more direct orthographic access to actual word meanings. This makes it a particularly apt language for investigating parafoveal semantic preview effects. Yan et al. (2009) found a semantic N+1 benefit for non-compound simplified Chinese character previews in Chinese reading. A semantic N+1 benefit has also been found with more complex compound characters in simplified Chinese reading (Yan et al., 2012; Yang et al., 2012), as well as in traditional Chinese reading (Tsai et al., 2012). Evidence for a semantic N+1 preview benefit has also been found in Korean, an alphabetic language with shallow orthography but using inter-word spaces (Yan et al., 2019).

The majority of what we know about parafoveal processing comes from native speakers of their first language (L1). In contrast, research investigating parafoveal processing in second language (L2) speakers is relatively sparse and has focused more on semantic processing. To our knowledge, there are only six studies investigating parafoveal processing in L2. Three of these focused primarily on semantic N+1 effects, one on orthographic and phonological N+1 effects, one on syntactic N+1 effects, and one on N+1 interference. Given that the current study investigates semantic N+1 effects, we discuss the semantic N+1 studies in depth and just briefly describe the three additional studies.

In the first GCB study, Altarriba et al. (2001) compared parafoveal processing in Spanish-English bilinguals who read sentences in both English and Spanish. The parafoveal mask in these sentences was manipulated to be either identical to the critical word (no display change), a translation of the critical word in the other language, or an unrelated word in the other language, as shown in Table 1 (columns). Moreover, as the rows in Table 1 indicate, materials were manipulated such that masks were either noncognate translations of the critical words (i.e., the masks shared the same meaning, but no orthography or phonology with the critical word), cognate translations of the critical words (i.e., masks and critical words were orthographically and phonologically similar), or pseudo-cognate translations of the critical words (i.e., orthographically similar, but semantically unrelated; *grasa* in Spanish means *grease* in English).^1^

**Table 1:** Example stimuli from Altarriba et al. (2001)

	Lead-In	English Critical Word	Related Spanish Mask	Unrelated Spanish Mask
Non-Cognate	The new brand of paper towel is	strong	fuerte	hambre
Cognate	The kitten was given a bowl of	cream	crema	torre
Pseudo-Cognate	Steve’s mom asked him to cut the	grass	grasa	falda

First-fixation duration and gaze duration results showed similar patterns: relative to an unrelated mask, there was a large N+1 preview benefit when the critical word was seen in the parafovea (no display change); interestingly, there was a similar benefit from cognate masks (*crema* changing to *cream*) and pseudo-cognate masks (*grasa* changing to *grass*), which did not differ from one another; most crucially, non-cognate masks (*fuerte* changing to *strong*) resulted in interference. This suggests that bilingual Spanish/English readers were able to make use of parafoveally available orthographic information (preview benefit from cognate and pseudo-cognate masks) but showed an interference from non-cognate masks (which were semantically but not orthographically related to the critical word).

Across two studies, Wang and colleagues (Wang et al., 2014; Wang et al., 2016) tested semantic parafoveal processing in Korean (L1) / Chinese (L2) bilinguals. In their first study Wang et al. (2014) had participants read Chinese (L2) sentences in which the critical word was parafoveally masked with an identical, orthographically related, phonologically related, semantically related, or unrelated mask. They found an N+1 preview benefit on the critical word only when the parafoveal mask was identical or orthographically related to the critical word. Interestingly, the authors also tested L2 reading proficiency and found that L2 speakers with higher proficiency showed a greater facilitation relative to those with lower proficiency. In their second study (Wang et al., 2016), participants again read sentences in Chinese. However, parafoveal masks were presented in Korean and were either cognate translation equivalents to the critical words, semantically related non-cognates, or unrelated to the critical words. This study showed an N+1 preview benefit from semantically related masks, both cognate and non-cognate, suggesting that L2 readers of Chinese are likely to extract semantic information parafoveally.

Jouravlev and Jared (2018) investigated N+1 effects across bilingual L1 Russian/L2 English speakers reading in English and varying Russian masks that were presented in the Cyrillic script. They found a phonological N+1 benefit even when there was no orthographic overlap between the Russian Cyrillic mask and the critical English word, and an orthographic N+1 benefit even if there was no phonological overlap between the Russian Cyrillic mask and the critical English word.

Vaughan-Evans et al. (2020) investigated N+1 effects across bilingual Welsh/English speakers reading in English and varying non-word masks that either adhered to Welsh syntactic rules or did not. They found that Welsh morphosyntactic rules were coactivated when reading in English. Due to its focus on morphosyntactic processing, this study differs quite markedly in theoretical scope from our present study (as is also reflected in the fact that Vaughan-Evans et al.’s morphosyntactic coactivation effects showed up in relatively “late” eye-tracking measures that are not considered here). We nevertheless mention it for completeness.

Finally, Fernandez et al. (2020) investigated the N+1 interference effect associated with uninformative masks in English with monolingual L1 English and bilingual L1 German/L2 English speakers. Both L1 and L2 readers showed the same graded pattern whereby N+1 interference became stronger with less “word-like” masks.

Potentially relevant to the current study is the fact that the frequency of letters and their combinations (e.g., bigram frequency, the frequency with which two adjacent letters occur within a language) can aid in language selection and word recognition for bilingual readers (e.g., Grainger & Beauvillain, 1987; Thomas & Allport, 2000; Vaid & Frenck-Mestre, 2002; Van Kesteren et al., 2012). It is indeed possible that the frequency of particular letter combinations in the masks used may aid in language selection and lexical access. However, the majority of this research has investigated single-word foveal processing of bilingual readers, and it remains to be seen how bigram frequencies may affect parafoveal processing during sentence reading. While Fernandez et al. (2020) did not directly investigate bigram frequency, they did use five types uninformative gaze-contingent masks that shared no features with the critical word (*rock*), two of which are particularly relevant: the “English-like” pseudo-word masks (*mish*) and “German-like” pseudo-word masks (*mand*, Schröter & Schroeder, 2018). They found that neither L1 English speakers nor L1 German/L2 English speakers were sensitive to the language-specificity of the pseudo-word masks (i.e., there were no differences between a target word that was masked with a more “German-like” pseudo-word or more “English-like” pseudo-word). This suggests that sensitivity to language-specific sub-lexical orthographic information may not arise in early stages of parafoveal processing, but rather emerge during later stages of lexical access or during foveal processing.

While the above six studies suggest that L2 readers are at minimum able to make use of visual/orthographic-level parafoveal information regardless of the L1/L2 language combination (as reflected in an N+1 orthographic preview benefit, and in N+1 interference when uninformative masks are used), it remains unclear whether they are able to process N+1 semantic information. It seems that for L2 readers, similar to L1 readers, N+1 semantic processing only occurs in some languages and under certain circumstances. For example, L2 readers of languages that map meaning more directly onto their orthography (like Chinese) seem able to extract semantic information parafoveally as well, at least when masks are presented in their L1. Given that there is a lack of research on L2 parafoveal processing, and that much of what has been done is investigating the somewhat elusive semantic N+1 benefit, in the current study we directly investigate orthographic and semantic processing by both L1 and L2 speakers of English.

## Current study

Given the general dearth of evidence on parafoveal processing in L2, our aim was to expand research in this area and investigate both orthographic and semantic parafoveal processing in L1 and L2 readers of English. Specifically, we tested a group of 51 native English speakers (L1 readers), and another group of 51 native German speakers (L2 readers) who were also proficient in English as established via an English proficiency test. Recall that previous research has found an N+1 semantic preview benefit for native German speakers tested in their L1, which is at least partly due to the clear spelling-to-sound correspondence in German (Hohenstein et al., 2010; Hohenstein & Kliegl, 2014). Our group comparison between L1 and L2 readers of English is therefore particularly interesting because the stronger spelling-to-sound correspondence in the native language (German) of our English L2 readers may lead to more efficient processing of parafoveally viewed German translation masks in those readers (even when they are reading in their more opaque second language, i.e., English). We used six different types of parafoveal mask for our GCB study: (1) a mask that was identical to the critical word of interest (no display change), (2) a non-word mask partial matched in orthography with the critical word (e.g., wook for the critical word wood), (3) a non-word mask matched in ascending and descending letters with the critical word (e.g., zwwl for the critical word wood), (4) a German non-cognate translation of the critical word (e.g., holz for wood), (5) a non-word mask partial matched in orthography with the German translation of the critical word (e.g., holn), and, finally, (6) a nonword string matched in ascending and descending letters with the German translation of the critical word (e.g., kxfs). The German non-cognate translation masks were taken from Friel and Kennison (2001), who ensured that the meanings of these translations could not be correctly guessed by native speakers of English without any prior exposure to German. It was therefore safe to assume that our L1 English participants would treat these non-cognate translations as non-words.

We had five hypotheses. Hypothesis 1: N+1 preview benefit not only from identical masks (“baseline,” no display change), but also from English orthography masks like wook (for the critical word wood) for both L1 and L2 readers, because orthographic N+1 preview benefits have been well established in previous research (e.g., Schotter et al., 2012). Hypothesis 2: N+1 interference effect for orthographically unrelated string masks compared to the previous two conditions, given that previous research has found that N+1 preview interference increases as a mask becomes less “word-like” (Fernandez et al., 2020; Vasilev & Angele, 2017). This should hold true for both L1 and L2 readers of our materials. Hypothesis 3: N+1 preview benefit when comparing the German translation mask with the identical mask (baseline) condition, but only for the L2 readers (whose L1 is German) and not for L1 readers (who are unfamiliar with German); this would corroborate previous research pointing to an N+1 semantic preview benefit (e.g., Hohenstein et al., 2010; Schotter, 2013; Veldre & Andrews, 2016; Wang et al., 2016). Hypothesis 4: equal N+1 parafoveal preview benefit when comparing the German orthography mask with the German translation mask condition, but again, only for L2 readers (whose L1 is German) and not for L1 readers (who are unfamiliar with German). Hypothesis 5: N+1 parafoveal interference effect for the German string mask condition in both groups (perhaps with a larger interference effect for L2 readers) given that they appear less word-like (Fernandez et al., 2020; Vasilev & Angele, 2017). In essence, we expected the following effect patterns per group (where “*a < b*” indicates the expectation of faster processing times for the critical word in masking condition *a* than in masking condition *b*) which should yield a *Group × Mask Condition* interaction in the overall analysis:

- L1 readers (unfamiliar with German)

$$ \mathrm{Identical}\ \left(\mathrm{baseline}\right)=\mathrm{English}\ \mathrm{orthography}<\mathrm{English}\ \mathrm{string}=\mathrm{German}\ \mathrm{translation}=\mathrm{German}\ \mathrm{orthography}=\mathrm{German}\ \mathrm{string} $$

- L2 readers (German L1):

$$ \mathrm{Identical}\ \left(\mathrm{baseline}\right)=\mathrm{English}\ \mathrm{orthography}=\mathrm{German}\ \mathrm{translation}=\mathrm{German}\ \mathrm{orthography}<\mathrm{English}\ \mathrm{string}=\mathrm{German}\ \mathrm{string} $$

The above is based on the assumption that bilingual L2 readers can parafoveally extract orthographic and semantic information from both L1 and L2 “in equal measures.” This admittedly rather idealistic assumption is only viable if the German native speakers in our study have reasonably high levels of proficiency and quality of lexical representation (as measured through spelling skill) in their English L2. We therefore administered an English spelling test as a measure of quality of lexical representation (Veldre & Andrews, 2014) and an English morphosyntax test as a measure of English proficiency (Wang et al., 2014; Whitford & Titone, 2015) to all of our participants, which enabled us to account for potential group differences.

## Method

### Participants

For the L1 group, 51 native speakers of English were recruited from the University of Glasgow student community. Participants in this group reported that they had no early (before the age of 6 years) exposure to a second language, and that they had no experience with German in particular. For the L2 group, 51 native speakers of German were recruited from Technische Universität Kaiserslautern in Germany. Participants in the L2 group all reported that they were late second-language learners of English, that they grew up in Germany (with only German spoken at home), and that they were not exposed to a second language before the age of 6 years. Their mean English acquisition age was 10.2 years (SD = 1.6 years; range: 6–15 years). None of our 102 participants reported to have a language or reading-related impairment, and all had normal or corrected-to-normal vision. Table 2 provides a summary of additional participant-related information per group. Both the mean English proficiency percentage out of 50 (Oxford Placement Test (OPT) – Part A) and mean spelling score are included as predictors in the statistical analysis.

**Table 2:** Participant information per group. Shown are numbers of males/females, average age in years, mean score on an English proficiency test (Oxford Placement Test (OPT) – Part A) and mean score in the English spelling test

L1	N Male, N Female	Mean (SD) Age	Mean (SD) OPT Score	Mean (SD) Spelling Score
English	14, 37	23.5 (4.1)	95.8 (4.3)	83.2 (8.1)
German	32, 19	25.0 (3.4)	79.4 (10.1)	77.1 (8.2)

### Materials

Sentences were presented in English. The experiment consisted of two eye-tracking studies, separated by a break (only the first study is reported here). The present experiment consisted of four practice trials, 24 critical items, and 24 filler trials. An example stimulus, and its associated parafoveal mask conditions, is shown in Table 3.

**Table 3:**
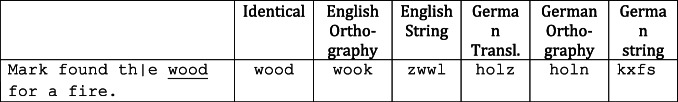
Example stimulus. Shown in the leftmost column is the sentence frame in which the critical word (here the underscored word wood) was embedded; “ | ” indicates the position of the invisible boundary at two character positions (ca. 1° of visual angle) to the left of the left edge of the critical word. Before crossing this boundary with an eye movement, the critical word was masked with one of six different types of letter strings (columns 2–7)

The sentence frames always started with a proper noun (e.g., *Mark*) or an article and a generic noun denoting a human or group of humans (e.g. *the child* or *the girls*), followed by a verb (e.g., *found*), the article *the* (with an invisible boundary being embedded between the characters ‘h’ and ‘e’), then one of the parafoveal masks (Table 3) respectively the critical word (e.g., *wood*) after crossing the invisible boundary, and finally, a spillover region which was always a three-word prepositional phrase (e.g., *for a fire*). No verb was used more than twice across the items.

The critical word and its German equivalent in the German translation mask condition were non-cognate pairs taken from Friel and Kennison (2001). As mentioned previously, these translations were unlikely to be correctly guessed by English L1 speakers unfamiliar with German. All masks were matched for length within items (but varied between four and 11 characters across items; mean length: 5.66 (SD:1.73)), and the English and German masks did not share the same first letter. We aimed to keep the orthography of our critical masks as “English” as possible (including in terms of capitalization) to ensure that any differences between language groups stem from language-specific lexical access (and not increased visual salience to unexpected English information). In German, all nouns are capitalized. However, given that a capitalized letter in a parafoveal word may increase attention to the parafoveal word for L1 English speakers (Rayner & Schotter, 2014) and that L1 German speakers are able to extract parafoveal semantic information regardless of capitalization (Hohenstein & Kliegl, 2014), all masks in the current study were presented completely in lower case. Additionally, any German masks that contained a letter that was not part of the English alphabet were alternatively presented; words with an umlaut were presented with an “e” following the vowel (e.g., Säule ➔ saeule (column); this occurred in four instances) and any words with an Eszett (ß) were instead presented with “ss” (e.g., Strauß ➔ strauss (bouquet); this occurred in two instances).^2^ Both types of changes are acceptable in German writing. See Appendix 1 for a complete list of items and masks.

The English and German orthography masks were created using the first three letters of the respective English/German mask, the remainder of the letters were replaced with matching descending/ascending letters to form a non-word (e.g., ). The English and German string masks were created by matching all the letters in the respective English/German mask with descending/ascending letters to form a non-word (e.g., ); there was no overlap in letters (in the corresponding letter position) between language-specific masks and the string masks. The letter shapes between the respective English and German masks (for both the orthographic and string conditions) were matched to minimize the detectable differences when the display change was made.

The critical words were of low expectancy, given their preceding sentence contexts. This was established using an offline sentence completion task taken by 23 native English speakers (who did not participate in the current study). Participants were provided with the beginning of a sentence (until the word prior to the critical word *wood* in Table 3) and were asked to complete the sentence with the first thing that came to their mind that was grammatically correct and made sense. Of the 23 participants * 24 items = 552 completions provided, only two (0.36%) matched the critical word of interest (across two different items). Thus, the sentence contexts were rather unpredictive of the critical words. Additionally, the critical words were high frequency; the mean log10 frequency was 1.65 (SD: 0.51) per million according to the Corpus of Contemporary American English (range: 0.52– 2.59). The German masks were also high frequency; the mean log10 frequency was 1.49 (SD: 0.50) per million according to the Ten Ten German Web Corpus (range: 0.36 – 2.31; Jakubíček et al., 2013).

While the materials were a one-way design with six levels (mask type conditions), overall the study was a 2 × 6 design, crossing the between-subjects/within-items factor *reading language* (L1, L2) with the within-subjects/within-items factor *mask type* (Identical, English orthography, English string, German translation, German orthography, German string). In order for us to test the influence of L2 semantic parafoveal processing we had to rigorously control our items. As outlined above, the critical masks were controlled for frequency and predictability, and the masks were also carefully designed such that the English and German translations did not share the first letter, did not share any orthographic information that may influence the L1 English speakers in identifying the German word (including false cognates), and were matched in length (after the orthographic changes). Due to these restrictions the number of items we were able to design was severely limited, leaving us with only 24 items that met all of the criteria. While this may limit the potential power of the study, we believe that the current study still makes important contributions to the field, and we encourage further research with different languages combinations that may yield more items.

### Apparatus

Our sample of native English speakers (L1 readers) was tested at the University of Glasgow, using an EyeLink 1000 desk mounted eye-tracker running at a sampling rate of 1,000 Hz. Participants’ heads were stabilized using a chin rest, and participants sat approximately 72 cm away from the monitor, such that two characters subtended about 1° of visual angle. Viewing was binocular, but only right-eye recordings were taken. Stimuli were presented on a Dell P1130 19-in. flat screen CRT with 1,024 × 768 pixel resolution, running at 150-Hz refresh rate. Display changes happened within around 6.20 ± 1.99 ms (mean ± SD) from the detection of an eye movement across the invisible boundary.

The sample of native German speakers (L2 readers) was tested at Technische Universität Kaiserslautern, using an Eyelink 1000 or an EyeLink Duo^3^ eye-tracker, both sampling at 1,000 Hz. Viewing was binocular, but only right-eye recordings were taken. Participants’ heads were stabilized using a chin rest, and participants sat approximately 90 cm away from the monitor, such that ca. 2.4 characters subtended 1° of visual angle. On both eye-trackers, stimuli were presented using a Samsung SyncMaster 959NF 19-in. flat screen CRT with 1,024 × 768 pixel resolution and running at 120-Hz refresh rate. Display changes happened within ca. 6.27 ± 2.42 ms (mean ± SD) from detecting an eye-movement crossing the invisible boundary.

### Procedure

The procedure was the same for both groups. The participant first completed a series of paper tasks: a language background questionnaire, the Oxford Placement Test (Part A) to assess English proficiency, and a misspelling identification task. This was followed by the two eye-tracking experiments. In its entirety, the study took approximately 60 min. Participants in Glasgow were paid £10 for their participation, and participants in Kaiserslautern were paid 10 € or given course credit for their participation.

The standard EyeLink 9-pt calibration procedure was used at the start of the eye-tracking task. Participants were recalibrated as deemed necessary by the experimenter, and there was an obligatory break (with subsequent recalibration) between the two eye-tracking experiments (of which only the first experiment is presented here). The study was self-paced, and participants were able to take a break (between trials) whenever needed; if a participant decided to take a break, they were recalibrated before continuing with the task. The study instructions were displayed on the stimulus display before the eye-tracking session started and also explained verbally by the experimenter. Participants were asked to read silently for comprehension and answer true/false comprehension questions that were presented immediately after each sentence. Comprehension questions were answered by pressing “x” for true and “m” for false on a Standard English keyboard with half of the statements being correctly answered with true and half correctly answered with false.

Each trial began with a drift correct (x coordinate: 58, y coordinate: 359) that corresponded to the first letter in the critical sentence. All sentences were presented in a monospaced font (Courier New) on one line in a font size of 19. Participants pressed the space bar when they were done reading the sentence. This was followed by a blank screen and the true/false comprehension statement (along with instructions to press “x” for true and “m” for false) centered on the screen in Courier New with a font size of 19. After inputting their answer, the drift correct screen would appear again and the participant could begin the next trial. Presentation order was randomly determined for each participant. When the first experiment was completed, participants were instructed to take a break and the experimenter would then prepare them for the second experiment (which is not reported here).

### Analysis

Overall question-answering accuracy was good (ca. 92%), indicating that participants were reading for comprehension. L2 readers were slightly less accurate (89%) than L1 readers (94%), but still clearly above chance performance. Accuracy was not considered further in subsequent analyses.

Prior to analysis of the eye-tracking data, we discarded 13.3% of trials in which either a *j-hook* occurred (i.e., a saccade crossed the invisible boundary, but landed on a word prior to the critical word region) or where a fixation landed in the critical word region *before* the display-change occurred (likely due to an eye-blink interfering with the display-change trigger). Given the increased potential of false positives when looking across several eye-movement measures an a priori decision was made to focus on three dependent variables that are important in parafoveal research^4^ (thus decreasing the likelihood of Type 1 error; see von der Malsburg & Angele, 2017).

Our analyses focused on two duration-based measures for the critical word region alongside the likelihood of skipping the critical word region. The two duration-based reading measures were *first fixation duration* (FFD) and *gaze duration* (GD). FFD is the duration (in ms) of the first fixation within the critical word region, and GD is the time (again, in ms) from the onset of the first fixation within the critical word region until the eye moves outside (either to the right or to the left) of the critical word region. Importantly, if the critical word region has been skipped during first-pass reading, both FFD and GD are scored as missing values. Prior to inferential analysis, FFDs and GDs below 80 ms or over 1,000 ms were removed (this affected 6.2% of the FFD data and 6.4% of the GD data in the L1 group, as well as10.4% of the FFD data and 11.3% of the GD data in the L2 group). In total, there were 1,046 valid data points for FFD and 1,044 for GD in the L1 group, as well as 862 valid data points for FFD and 853 for GD in the L2 group.

As a complementary measure, we analyzed the percentage of trials where the critical word region was skipped during first-pass reading (skipping rate). This measure is binary for any given trial (1 if the critical word region was skipped, 0 otherwise) and considers more valid cases than the duration measures (no data exclusion other than excluding trials with j-hooks or erroneous display-change triggers).

The data were inferentially analyzed using generalized linear mixed-effects models (GLMM), as implemented in the *lme4* package (Bates et al., 2019) in R (R Core Team, 2019). *P*-values were determined via the *lmerTest* package (Kuznetsova et al., 2019). For analyses of the continuous duration variables, FFD and GD, we assumed a Gamma distribution with identity link in the family arguments of the models. This accounts for the positive skew in the distribution of duration data while maintaining additive relationships between dependent and independent variables (see Lo & Andrews, 2015).

In all models, fixed effects included the factors Group (2 levels: L1, L2), Mask Type (six levels: Identical, English Orthography, English String, German Translation, German Orthography, German String), and the Group × Mask Type interaction. For each model the random-effects structure was maximally specified (Barr et al., 2013), but in all cases failed to converge. Therefore, we ran effect-specific maximal models with random slopes specified for the comparison in question; see Appendix 2 for model syntax and output. In all models, we included the participant-specific covariates Spelling Score and OPT Score as standardized continuous control predictors.^5^

Based on our a priori hypotheses, a custom contrast matrix was built with five comparisons: (1) Identical versus English orthography, (2) English orthography versus English string, (3) Identical versus German, (4) German versus German orthography, and (5) German orthography versus German string (see statistical code for hypothesis matrix and contrast matrix). Sum contrast coding was used for language (-.5/.5). Given that a priori contrasts have been argued to be more useful than omnibus F-tests, no additional analyses were run (see Schad et al., 2020). Below, estimates, *t*, and *p* values are reported; see Table 4 for mean values and standard deviations.^6^ Data and statistical code are available at https://osf.io/ujpkt/.

**Table 4:** Mean values and 95% confidence intervals per measure and condition

L1 English						
	FFD (ms)		GD (ms)		Skipping (%)	
Condition	M	95% CI	M	95% CI	M	95% CI
Identical	207	[197, 218]	246	[229, 263]	7.5	[3.7, 11.3]
English Orthography	207	[196, 217]	248	[231, 264]	6.0	[2.5, 9.4]
English String	214	[204, 224]	260	[243, 277]	5.9	[2.5, 9.2]
German	218	[206, 230]	264	[247, 281]	5.9	[2.5, 9.2]
German Orthography	214	[203, 225]	256	[240, 271]	5.4	[2.1, 8.7]
German String	223	[213, 235]	271	[253, 289]	6.0	[2.6, 9.5]
L2 English (L1 German)						
	FFD (ms)		GD (ms)		Skipping (%)	
Condition	M	95% CI	M	95% CI	M	95% CI
Identical	217	[206, 227]	284	[262, 306]	10.3	[5.7, 15.0]
English Orthography	235	[220, 249]	309	[28, 331]	11.8	[6.8, 16.8]
English String	244	[227, 260]	317	[291, 343]	7.4	[3.2, 11.6]
German	256	[242, 272]	338	[313, 362]	7.6	[3.6, 11.6]
German Orthography	225	[208 , 234]	288	[264, 312]	10.8	[5.9, 15.6]
German String	256	[234, 268]	334	[305, 363]	12.0	[6.9, 17.1]

*FFD* First-Fixation Duration, *GD* Gaze Duration, *Skipping* Skipping rate

## Results

### First-fixation duration

In terms of main effects for first-fixation duration (FFD), the German mask condition evoked a greater FFD than the identical mask condition (hypothesis 3; est.= 23.53, *t*=3.12, *p*=0.002), the German mask condition also evoked a greater FFD than the German orthography mask condition (hypothesis 4; est.= -16.33, *t*=-2.22, *p*=0.027), and the German string mask condition evoked a greater FFD than the German orthography mask condition (hypothesis 5; est.= 18.17, *t*=2.31, *p*=0.021). There was a difference between L1 and L2 speakers (est.= -22.98, *t*=-2.52, *p*=0.012), with L2 speakers having a greater overall FFD.

**Fig. 2:**
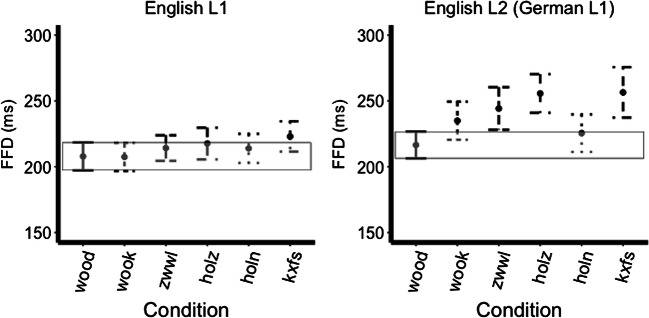
First fixation duration across mask types, bars represent 95% confidence intervals (the box encompasses the confidence interval of the identical level)

There was an interaction between language and identical versus German mask conditions (hypothesis 3; est.=-26.63, *t*=-2.05, *p*=0.034), with the German mask condition evoking a greater FFD than the identical mask condition but only for the L2 speakers. There was an interaction between language and German versus German orthography mask conditions (hypothesis 4; est.= 28.48, *t*=2.19, *p*=0.029), with the German mask condition evoking greater FFD than the German orthography mask condition but again only for the L2 group. Nothing else reached significance (all *ps* >0.05; see Fig. 2).

### Gaze duration

In terms of main effects for gaze duration (GD), the German mask condition evoked a greater GD than the identical mask condition (hypothesis 3; est.= 36.77, t=3.36, *p*<0.001), the German mask condition evoked a great GD than the German orthography mask condition (hypothesis 4; est.= -24.19, *t*=-2.16, *p*=0.031), and the German string mask condition evoked a greater GD than the German orthography mask condition (hypothesis 5; est.= 24.92, *t*=1.93, *p*=0.05). There was a difference between L1 and L2 speakers (est.= -57.64, *t*=-3.90, *p*<0.001), with L2 speakers having a greater overall GD.

**Fig. 3:**
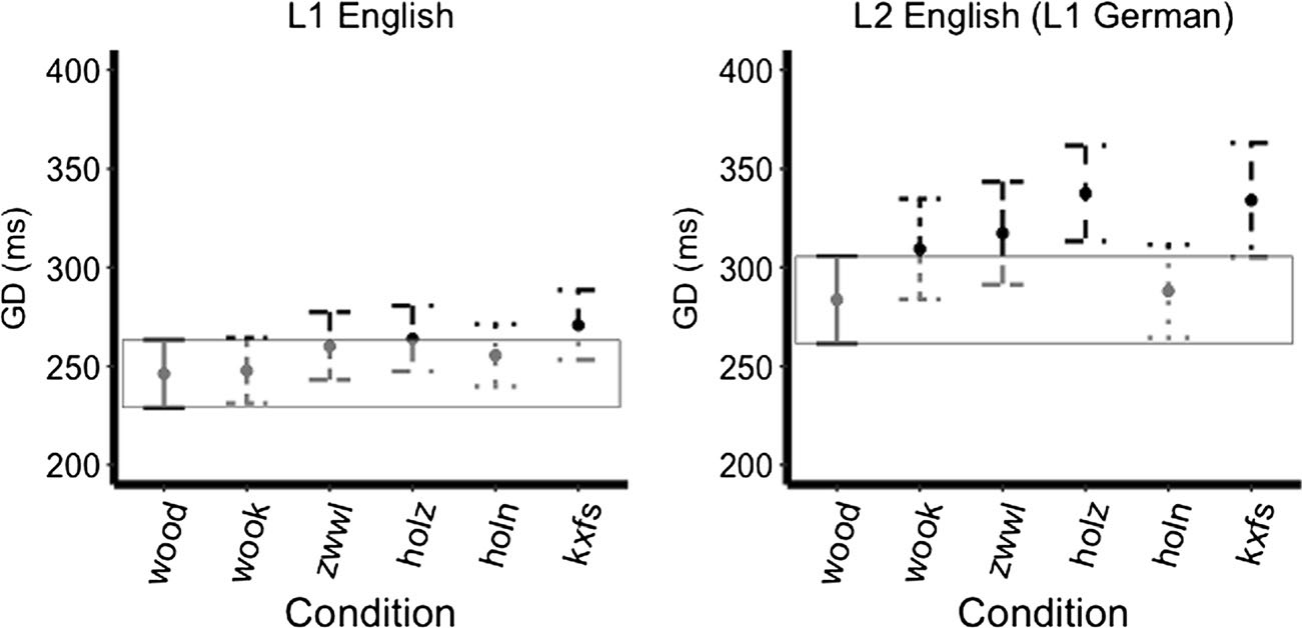
Gaze duration across mask types, bars represent 95% confidence intervals (the box encompasses the confidence interval of the identical level)

There was also an interaction between Language and German versus German orthography mask conditions (hypothesis 4; est.=44.71, *t*=2.20, *p*=0.028), with the German mask condition evoking a greater GD than the identical mask condition across both groups. Nothing else reached significance (all *ps* >0.05; see Fig. 3).

### Skipping rate

**Fig. 4:**
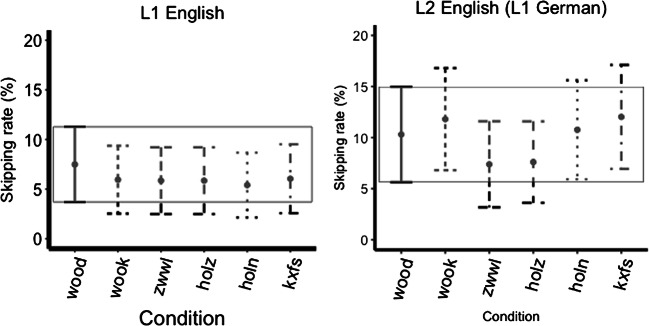
Skipping rate in percentage across mask types, bars represent 95% confidence intervals (the box encompasses the confidence interval of the identical level)

In terms of main effects for skipping rate, the English orthography mask condition was skipped more than the English string mask condition (hypothesis 2; est.= -1.64, *t*=-2.94, *p*=0.003), and the identical mask condition was skipped more than the German mask condition (hypothesis 3; est.=-0.93, t=-2.22, *p*=0.027). Nothing else reached significance (all *ps* >0.05; see Fig. 4).

## Discussion

In this study we investigated parafoveal processing by L1 and L2 speakers of English (with an L1 of German) while reading in English. Despite the reality that more than half of the world is bilingual (Marian & Shook, 2012), the majority of research investigating parafoveal processing has been conducted with L1 speakers. We were interested in whether L2 speakers were able to make use of semantic and orthographic information parafoveally. As outlined above, we manipulated six parafoveal masks: an identical word ( ), an English orthography mask ( ), an English string mask ( ), a German translation equivalent mask ( ), a German orthography mask ( ), and a German string mask ( ).

Our first hypothesis was that both L1 and L2 speakers would have an N+1 benefit when the mask was orthographically related to the target word (comparing  to ). Our results suggest that this is indeed the case since there were no differences in the FFD, GD, or skipping rate between the identical ( ) and English orthography ( ) mask conditions for either group. This suggests that both groups derived a similar benefit whether  or  was in the parafoveal area. This finding is in line with previous literature that readers have an orthographic N+1 benefit for masks that share some orthographic overlap with the critical word (e.g., L1: see Schotter et al., 2012; L2: Altarriba et al., 2001; Wang et al., 2014; Wang et al., 2016).

Our second hypothesis was an N+1 interference on the critical word in the context of the English string mask ( ) relative to the English orthography mask ( ) for both L1 and L2 speakers, given that research has found that the less word-like a parafoveal mask becomes, the greater the interference (e.g., Vasilev & Angele, 2017). Contrary to our hypothesis, we found no differences between these conditions for either group in our duration measures (FFD and GD). However, we did find a difference in skipping rate, with the critical word in the English orthography condition being skipped more than in the English string condition for both groups. This suggests that both groups were more likely to skip the critical word when the mask and the critical word share the first three letters. The lack of differences in duration measures may be due in part to the fact that our string mask matched the shape of the critical word, making the switch between the mask and critical word less noticeable. It may be that noticeable changes between parafoveal mask and critical word impact N+1 effects (e.g., Slattery et al., 2011).

Our third hypothesis tested whether there was a semantic benefit from the German masks for L2 speakers (i.e., non-cognate translation,  compared to ). Research investigating semantic N+1 benefits have been mixed (see Schotter et al., 2012), but have been found to exist for German speakers reading in German (Hohenstein et al., 2010; Hohenstein & Kliegl, 2014), for readers of languages with a tight mapping between orthography and meaning (*Chinese:* Tsai et al., 2012; Yan et al., 2009; Yan et al., 2012; Yang et al., 2012; *Korean:* Yan et al., 2019) and in English, when synonyms were used (Schotter, 2013). A semantic N+1 benefit has also been found for L2 readers of Chinese (L1 Korean) when the parafoveal mask was presented in Korean (Wang et al., 2016). Additionally, masked translation priming lexical decision tasks show a translation priming effect when an L1 word serves as a prime for an L2 target word (see Altarriba & Basinight-Brown, 2007; Duñabeitia et al., 2010). Therefore, we hypothesized an N+1 benefit for L2 speakers of English (L1 German) when a German translation serves as the mask for an English word, and a N+1 interference for L1 speakers (they should treat it as a non-word).

We found differences in FFD, GD, and skipping rate between the German mask and the identical mask conditions, but not in the direction predicted. For the duration measures, the German mask condition evoked greater reading durations than the identical mask condition but only for the L2 group; there was no difference between the measures for L1 speakers. For skipping measures, more skipping of the critical word occurred in the identical word mask condition than in the German mask condition. As evident in Figs. 2 and 3, the German mask condition evoked the greatest reading times for the L2 speakers, suggesting a large N+1 interference rather than an N+1 benefit. It is possible that this interference stems from some sort of switching cost incurred by L2 readers when switching from English to German and back to English. We discuss this below.

The fourth hypothesis was that there would be an orthographic N+1 benefit for L2 speakers in the German word mask condition as compared to the German orthography mask condition (  compared to ), but no difference for L1 speakers. While we indeed found an N+1 benefit for L2 speakers in terms of reading durations, and no difference for L1 speakers, we would argue that the differences may not be due to the reasons we hypothesized. Given that there was quite a large N+1 interference for L2 speakers for the German mask condition, it stands to reason that the same type of interference would impact the German orthography mask condition. However, we find a clear benefit. We return to this shortly.

Lastly, we compared the German orthography mask and the German string mask conditions (  vs. ). We hypothesized an N+1 interference for both L1 and L2 groups, but a potentially larger interference for the L2 group (given that the mask becomes less word-like for L2 speakers only). Both groups show this difference in the GD, suggesting that both groups indeed show interference from the German string mask condition.

In terms of individual differences, we included a measure of quality of lexical representation (via a test for spelling) and an English proficiency (via a test for English morphosyntax), both of which have previously been found to impact the efficiency in which parafoveal information is extracted (e.g., Veldre & Andrews, 2014; Wang et al., 2014; Whitford & Titone, 2015). Due to convergence reasons, we were not able to include control-predictor related random slopes (see Barr et al., 2013, who have reported that it may not be essential to include control predictors in random effects), and as a result believe that only tentative interpretation is warranted. We found a significant effect of spelling and no impact of proficiency across almost all models. Previous research with L1 speakers has assessed quality of lexical representation via spelling and reading skills (Veldre & Andrews, 2014), while L2 research has assessed quality of lexical representation via L2 exposure. The current study suggests that spelling skills may be an appropriate measure of quality of lexical representation for both L1 and L2 speakers, but we suggest further research.

### Bilingual models of word recognition

The main aim of the current study was to test the somewhat elusive semantic parafoveal N+1 benefit by testing late L2 speakers of English (with a German L1) using non-cognate translation equivalent parafoveal masks during English sentence reading. We hypothesized a semantic N+1 benefit for the L2 speakers when reading the English word  after parafoveally viewing the German translation mask , and a potential orthographic benefit reading  after parafoveally viewing  (which shares the first three characters with the German translation). We found an N+1 interference following  and a benefit following . These findings are unexpected and seemingly contradictory. The current study was set within the framework of the semantic N+1 preview benefit. However, these findings may be better explained within models of bilingual word recognition though this was not the aim of the particular study.

The Bilingual Interactive Activation Plus (BIA+) model of bilingual word recognition (Dijkstra & Van Heuven, 2002) argues that the bilingual lexicon is integrated between languages and is language non-selective. When recognizing a word at an orthographic level, orthographic candidates are accessed in parallel, and the overlap between the orthography/word and the lexical representation determines the activation (as opposed to the language node to which the word belongs); the more overlap between the orthographic string and its representation, the more active it becomes. It may be that when parafoveally processing the complete German word , the German word is strongly activated which in turn activates the German language node, and inhibits words from the English language node. A switching cost is incurred when activating the German word but ultimately fixating on the English word. In a study using both self-paced reading and EEG, Litcofsky and Van Hell (2017) found that switching from an L1 to an L2 (stronger to weaker language) incurred costs related to restructuring at a sentence level, and switching from an L2 to an L1 (weaker to stronger language) incurred costs related to the previous suppression of the stronger L1. Given that in the current study there is a switch from L2 to L1 back to L2, it is possible that switching costs were compounded, leading to an N+1 interference.

When processing the partial German orthographic information  in the parafoveal area, both the English and the German lexicon remain active, given that holn is neither a true German word nor an English word. We would argue that the orthographic overlap ( ) narrows the lexical and semantic candidates in both English and German, and when reading  the reader is able to access the semantic representation quickly via its heightened German activation. Given that English also remains active, no (or less) switching cost is incurred. This, in turn, leads to an N+1 facilitation. This would mean that the N+1 benefit we see here is both an orthographic and a semantic facilitation. It is important to note that in language switching research, a word in a list, or a word or part of a sentence, switches from one language to the other for the duration of the trial. In the current study this is not the case: a word may cause a switch between languages, but the switch does not remain for the entirety of the presentation; rather it is available only parafoveally (the reader never directly fixates on the L2 word). Further research is needed to determine the extent to which the above-mentioned results from language switching research apply to parafoveal language switches.

The research discussed above focuses on single word recognition in bilinguals. However, research on bilingual sentence processing also offers insights regarding language activation. This research suggests that both languages may be activated in sentence reading for bilinguals, even in cases when there is a strong semantic context in one of the two languages (see van Assche et al., 2012, for a review). Given that the items in our study were designed to have a weak semantic constraint, it is even more likely that both languages remained activated during reading. This reasoning provides more support for our suggestion above that the unexpected interference effect with the German translation mask may derive from switching costs.

Our research also contributes to the work on word recognition within sentences by extending the types of words investigated. Most of the prior research on bilingual word recognition within sentences has focused on cognates, non-identical cognates, and interlingual homographs (e.g., Duyck et al., 2007; Elston-Güttler et al., 2005; Schwartz & Kroll, 2006). Indeed, Van Assche et al. (2011) found that the more orthographic overlap between Dutch and English cognates (identical: ring/ring, non-identical: shoulder/schouder) the more reading time benefit elicited in both high and low semantic constraint English sentences by L2 English speakers (L1 Dutch). The current study, in contrast, focuses on non-cognate translations. It provides the first evidence that there is a benefit for L2 speakers in sentence reading for *non-cognate* orthographic information in the parafovea, but only when there is partial orthographic overlap.

Another line of research may be relevant for the current study; research with masked translation priming lexical decision tasks has shown that there is a translation priming effect when an L1 word (both cognate and non-cognate translations) serves as the prime for an L2 target word (for reviews, see Altarriba & Basinight-Brown, 2007; Duñabeitia et al., 2010), and even for an L3 target word (Aparicio & Lavaur, 2016). However, we hesitate to draw too many parallels between these two paradigms given that the tasks are very different (lexical decision vs. reading for comprehension); the lexical decision paradigm involves single-word recognition and the GCB paradigm involves reading sentences. However, it is possible that this type of priming may occur using the GCB paradigm employed here, given that in both paradigms the participant is exposed to a word (prime or parafoveal mask) for a short duration that they are not typically aware of.

### Limitations and future directions

We believe the main limitation of the current study is the relatively low number of items per condition, even with the relatively high participant sample. We took extreme care to control the critical English world in terms of predictability, frequency, and length. In particular, the English critical word and its German non-cognate translation never shared the first letter, the two words were exactly the same length, and importantly the German translation provided no helpful information to the monolingual English speakers (i.e., the English meaning could not be guessed from the German word, as judged by 250 monolingual English speakers). This made the materials particularly well suited to make comparisons across items and to test the impact of L1 orthographic and semantic information during L2 reading. As a direct result, however, the number of potential items was quite limited, and this may impact the overall power of the study.

Despite this potential limitation, we believe that the current study has important implications for future research and for models of bilingual and monolingual reading and comprehension. To the knowledge of the authors, there is no mention of parafoveal processing in any model of L2 processing/reading. It remains to be seen how these findings will fit into the framework of current bilingual word recognition theories, L2 reading models, and even within L1 reading models (which do attempt to explain parafoveal processing). It points to several open questions that will likely be fruitful avenues to pursue, including the following. How does the BIA+ Model explain N+1 effects? Is it possible to have an N+1 facilitative effect from orthography and an interference effect from semantics from L1 words when reading in an L2? Is it possible that switching costs can be incurred parafoveally, even without conscious awareness of the language of parafoveal word? How do models of L1 reading (that do discuss parafoveal research) account for this L2 pattern of results?

#### Conclusions

The current study adds to the limited research that has investigated parafoveal processing in L2 speakers. Not only was semantic information tested via non-cognate translations, but orthographic information was also tested in both English and German. We found that both L1 and L2 speakers derive a benefit from English orthographic masks, which suggests that L2 speakers are able to access at least some visual/orthographic information from their L2 parafoveally. L2 speakers of English did not seem to derive a benefit (rather an interference) when a non-cognate translation mask from their L1 (German) was viewed parafoveally, but did derive a benefit from an orthographic mask related to the non-cognate translation. While unexpected, it may be that L2 speakers incur a switching cost when the complete German word is presented parafoveally, and are able to derive a benefit by keeping both lexicons active when a partial German word is presented parafoveally (which narrows down lexical candidates). If this is the case, then this would be the first evidence of a combination orthography/semantic N+1 benefit, suggesting that these factors may interact in some way during reading. This would also be the first evidence of a semantic N+1 effect by L2 speakers reading in an alphabetic script.

## Appendix 1

English critical items and masks [Identical, English orthography, English string, German translation, German orthography, German string].

## Appendix 2 Linear mixed effects model syntax and output.

### First-fixation duration

#### Language-specific maximal model

glmer(FIRST_FIXATION_DURATION ~ 1 +Condition*Language + covariate_spell + covariate_OPT + (1 | Participant) + (1 + Language| item), data=data_frame, family = Gamma(link = "identity")

**Table:**

	Dependent Variable			
Predictors	Estimates	SE	Statistic	p
**(Intercept)**	**226.04**	**3.42**	**66.02**	**p<0.001**
**English**	**-22.98**	**9.13**	**-2.52**	**p<0.05**
**Spelling**	**-0.85**	**0.42**	**-2.02**	**p<0.05**
Proficiency	0.56	0.91	0.62	0.54

#### Condition-specific maximal model

glmer(FIRST_FIXATION_DURATION ~ 1 + Condition*Language + covariate_spell + covariate_OPT + (1 + Condition | Participant) + (1 + Condition | item), data=data_frame, family = Gamma(link = "identity")

**Table:**

	Dependent Variable			
Predictors	Estimates	SE	Statistic	p
**(Intercept)**	**222.81**	**3.21**	**69.44**	**p<0.001**
Identical vs. Eng. orthography	6.82	6.63	1.03	0.30
Eng. orthography vs. Eng. string	7.78	7.54	1.03	0.30
**Identical vs. German**	**23.53**	**7.55**	**3.12**	**p<0.01**
**German vs. Ger. orthography**	**-16.33**	**7.37**	**-2.22**	**p<0.05**
**Ger. orthography vs. Ger. string**	**18.17**	**7.88**	**2.31**	**p<0.05**
**Spelling**	-0.70	0.40	-1.75	0.08
Proficiency	0.69	0.86	0.80	0.42

#### Condition*Language -specific maximal model

glmer(FIRST_FIXATION_DURATION ~ 1 + Condition *Language + covariate_spell + covariate_OPT + (1 | Participant) + (1 + Condition short : Language | item), data=data_frame, family = Gamma(link = "identity")

**Table:**

	Dependent Variable			
Predictors	Estimates	SE	Statistic	p
**(Intercept)**	**225.76**	**3.31**	**68.23**	**p<0.001**
**Spelling**	**-0.84**	**0.41**	**-2.08**	**p<0.05**
Proficiency	0.54	0.88	0.61	0.55
Identical vs. Eng. orthography: English	-13.73	11.37	-1.21	0.23
Eng. orthography vs. Eng. string: English	1.36	12.38	0.11	0.91
**Identical vs. German: English**	**-26.63**	**12.96**	**-2.05**	**p<0.05**
**German vs. Ger. orthography: English**	**28.48**	**13.03**	**2.19**	**p<0.05**
Ger. orthography vs. Ger. string: English	-22.25	13.20	-1.69	0.09

### Gaze duration

#### Language-specific maximal model

glmer(GAZE_DURATION ~ 1 + Condition *Language + covariate_spell + covariate_OPT + (1 | Participant) + (1 + Language| item), data=data_frame, family = Gamma(link = "identity")

**Table:**

	Dependent Variable			
Predictors	Estimates	SE	Statistic	p
**(Intercept)**	**285.89**	**6.65**	**43.00**	**p<0.001**
**English**	**-57.64**	**14.79**	**-3.90**	**p<0.001**
**Spelling**	**-1.84**	**0.66**	**-2.78**	**p<0.01**
Proficiency	1.54	1.45	1.06	0.29

#### Condition-specific maximal model

glmer(GAZE_DURATION ~ 1 + Condition *Language + covariate_spell + covariate_OPT + (1 + Condition | Participant) + (1 + Condition | item), data=data_frame, family = Gamma(link = "identity")

**Table:**

	Dependent Variable			
Predictors	Estimates	SE	Statistic	p
**(Intercept)**	**275.83**	**6.13**	**45.01**	**p<0.001**
Identical vs. Eng. orthography	14.99	11.72	1.28	0.20
Eng. orthography vs. Eng. string	12.95	8.89	1.46	0.15
**Identical vs. German**	**36.77**	**10.96**	**3.36**	**p<0.001**
**German vs. Ger. orthography**	**-24.19**	**11.20**	**-2.16**	**p<0.05**
**Ger. orthography vs. Ger. string**	**24.92**	**12.93**	**1.93**	**p=0.05**
**Spelling**	**-2.08**	**0.64**	**-3.27**	**p<0.01**
Proficiency	2.52	1.39	1.81	0.07

#### Condition*Language -specific maximal model

glmer(GAZE_DURATION ~ 1 + Condition*Language + covariate_spell + covariate_OPT + (1 | Participant) + (1 + Condition: Language | item), data=data_frame, family = Gamma(link = "identity")

**Table:**

	Dependent Variable			
Predictors	Estimates	SE	Statistic	p
**(Intercept)**	**292.47**	**6.45**	**45.32**	**p<0.001**
**Spelling**	**-1.87**	**0.65**	**-2.88**	**p<0.01**
Proficiency	1.34	1.43	0.94	0.35
Identical vs. Eng. orthography: English	-23.10	18.43	-1.25	0.21
Eng. orthography vs. Eng. string: English	16.67	19.64	0.85	0.40
Identical vs. German: English	-27.95	21.43	-1.31	0.19
**German vs. Ger. orthography: English**	**44.71**	**20.35**	**2.20**	**p<0.05**
Ger. orthography vs. Ger. string: English	-37.59	24.10	-1.56	0.12

### Skipping

#### Language-specific maximal model

glmer(Skipping ~ 1 + Condition*Language + covariate_spell + covariate_OPT + (1 | Participant) + (1 + Language| item), data=data_frame, family = binomial)

**Table:**

	Dependent Variable			
Predictors	Estimates	SE	Statistic	p
**(Intercept)**	**-0.38**	**0.60**	**-0.64**	**p<0.001**
English	-0.38	0.60	-0.64	0.52
Spelling	0.00	0.03	0.15	0.88
Proficiency	-0.05	0.06	-0.81	0.42

#### Condition-specific maximal model

glmer(Skipping ~ 1 + Condition*Language + covariate_spell + covariate_OPT +

(1 + Condition | Participant) + (1 + Condition | item), data= data_frame, family = binomial)

**Table:**

	Dependent Variable			
Predictors	Estimates	SE	Statistic	p
**(Intercept)**	**-4.30**	**0.27**	**-15.66**	**p<0.001**
Identical vs. Eng. orthography	0.01	0.44	0.02	0.98
**Eng. orthography vs. Eng. string**	**-1.64**	**0.56**	**-2.94**	**p<0.01**
**Identical vs. German**	**-0.93**	**0.42**	**-2.22**	**p<0.05**
German vs. Ger. orthography	0.45	0.38	1.21	0.23
Ger. orthography vs. Ger. string	-1.07	0.56	-1.90	0.06
Spelling	0.00	0.03	-0.12	0.91
Proficiency	-0.02	0.07	-0.29	0.77

#### Condition*Language -specific maximal model

glmer(Skipping ~ 1 + Condition*Language + covariate_spell + covariate_OPT +

(1 | Participant) + (1 + Condition: Language | item), data= data_frame, family = binomial)

**Table:**

	Dependent Variable			
Predictors	Estimates	SE	Statistic	p
**(Intercept)**	**-4.02**	**0.26**	**-15.60**	**p<0.001**
**Spelling**	0.00	0.03	0.08	0.94
Proficiency	-0.05	0.07	-0.72	0.47
Identical vs. Eng. orthography: English	-0.57	0.75	-0.76	0.45
Eng. orthography vs. Eng. string: English	0.83	0.81	1.02	0.31
Identical vs. German: English	0.18	0.79	0.23	0.82
German vs. Ger. orthography: English	-0.72	0.77	-0.95	0.34
Ger. orthography vs. Ger. string: English	0.01	0.81	0.01	0.99

## Footnotes

1 Note that the design was not fully factorial. The authors report additional independent group analyses that provide results similar to the “matched analysis” referred to here.

2 Note that mask length matching was done after making letter changes.

3 Due to a malfunction in the host computer the Eyelink 1000 was replaced by an Eyelink Duo. The program, setup, display computer, display screen, and room remained the same across the entirety of the study. Twenty participants were run with the Eyelink Duo while 31 participants were run using the Eyelink1000. FFD, GD, and skipping rate did not significantly differ across the two machines (all *ps* > 0.19).

4 While we did not have particular a priori hypotheses about single fixation duration and total duration, we have included the values in the stored data for the interested reader.

5 To ensure that mask length did not impact the overall results for the duration measures, we additionally ran the models with the centered covariate of length. Overall, the pattern of results remained the same, suggesting that the impact of the fixed effects was independent of word length.

6 To ensure that the traditional N+1 effect was detectable in the current study, we additionally ran a LMER on gaze duration and first fixation duration using treatment contrasts with the Identical mask serving as the baseline. We found that for both measures the English String mask condition evoked greater duration than the Identical mask condition (FFD: est.= 13.74, t=2.03, p=0.042 /GD: est.= 24.49, t=2.20, p=0.027).
